# Can the implementation of family doctor contracted service enable the elderly to utilize primary health care services more equally? empirical evidence from Shandong, China

**DOI:** 10.1186/s12875-022-01630-0

**Published:** 2022-02-21

**Authors:** Shusheng Huang, Aitian Yin, Qianqian Liu, Xihong Sun

**Affiliations:** 1 Center of Health Management and Policy Research, School of Public Health, Cheeloo College of Medicine, Shandong University, No. 44 Wenhua West Road, Lixia District, Jinan, 250012 Shandong China; 2 Student Counseling Center, Shandong University, Jinan, 250012 China

**Keywords:** elderly population, inequality, family doctor contracted service, concentration index, China

## Abstract

**Background:**

While the elderly are facing greater health risks, they also face more serious inequalities in utilization of medical services. The family doctor contracted service is the core policy of the Chinese government to cope with aging and to achieve the outcome that everyone has the right to primary health care. However, previous research has neither revealed the degree of inequality in the use of contracted services among the elderly in China, nor has it revealed what factors are related to the inequality in the use of services.

**Objective:**

Assess and decompose the inequality in the use of family doctors contracted services in the elderly population in China.

**Methods:**

A cross-sectional study of 1037 elderly people was conducted in Shandong Province, China. According to the first consultation rate of family doctors, the physical examination rate, the healthy lifestyle guidance rate and the chronic disease management rate, the situation of elderly people’s utilization of family doctor contracted service was investigated. The concentration index is used to measure the degree of inequality in the use of family doctors contracted services by the elderly. In order to test the contribution of different factors to the inequality of utilization of family doctors contracted services, the concentration index was also decomposed.

**Results:**

The first consultation rate of family doctors for the elderly in Shandong Province was 24.6%, the physical examination rate was 65.8%, the healthy lifestyle guidance rate was 13.7%, and the chronic disease management rate was 52.2%. The horizontal inequality index of the healthy lifestyle guidance rate and the chronic disease management rate were 0.451 and 0.573, respectively, indicating that there is an inequality of pro-wealth. The concentration index of physical examination rate and chronic disease management rate is negative (− 0.260, − 0.518), which means inequality to the poor. Education level is the most important factor affecting the unequal utilization of health services for the elderly, followed by income.

**Conclusion:**

The family doctor contracted service has had a positive impact on alleviating the health inequality in the utilization of basic medical and health services for the elderly. Although there is still inequality in terms of pro-wealth for the elderly, the utilization of family doctor contracted service has weakened the inequality of service utilization brought about by income. Enhancing the health literacy of the elderly, narrowing the gap between the rich and the poor, bridging the gap between urban and rural areas, and building a harmonious family relationship can promote the realization of basic medical and health care services for every elderly.

## Introduction

In line with global trends, China’s population aging process is also accelerating [1]. As of the end of 2019, the number of elderly people over 60 years old in China has reached 254 million, accounting for 18.1% of the total population [2]. The elderly is not only a special group with increasing health vulnerability and disease risk, but also face more serious inequality in utilization of medical and health services. It causes the elderly, especially the low-income elderly, to fall into the vicious circle of health poverty and income poverty due to inequality in utilization of medical and health services, and therefore face greater health risks [3].

In 2016, China has begun to establish and implement a family doctor contracted service system. The expected goal of the system is to provide more equitable and accessible primary health care services for people, eliminate inequalities in the utilization of basic medical and health services, and achieve the goal of health for all [4]. The service provider of the family doctor contracted service is a team of general practitioners. The team usually consists of general practitioners, public health doctors and nurses. Residents voluntarily sign service agreements with a team of family doctors of their free choice. After signing the contract, residents can choose the primary medical and health institution where the family doctor is located for the first consultation. The family doctor team provides residents with basic medical services, basic public health services and personalized health management services [5]. In the content of family doctor contracted service, services for the elderly include physical examination, lifestyle and health status assessment, provision of personalized health management plans, healthy lifestyle guidance, and standardized management services for chronic diseases. As of 2020, the coverage of China’s family doctors contracted service has exceeded 85%, and the coverage of family doctor contracted service for the elderly has exceeded 95% [6], basically achieving full coverage of the elderly. In the current situation of China’s increasingly aging population, the level of equality in the utilization of basic medical and health services for the elderly is of great significance for China to achieve basic medical and health services for everyone, and to smoothly pass the peak of population aging. Family doctor contracted service is the core policy of the Chinese government to deal with the issue of aging. Does the family doctor contracted service system play its due role in improving the inequality in the utilization of basic medical and health services for the elderly? If there is inequality, what are the factors that affect the equality of the elderly in utilizing basic medical and health services? Answering these questions is conducive to evaluating the effectiveness of family doctor contracted service and adopting corresponding improvement measures to make them play a greater health gain effect.

Previous studies on inequality of utilization of medical services mostly examined the inequality and influencing factors of different social demographic and economic characteristics in terms of physical function, psychological status, and self-evaluation of health condition [7–9]. It is essentially a kind of social inequality, that is, health inequality with systemic differences between social groups with different advantages [10]. Although previous studies have proved that there is a continuous and stable connection among gender, age, marriage, education, income and utilization of medical services [11], few studies have involved inequality in utilization of medical services among homogeneous groups, that is, under the premise of weakening the influence of socio-economic status on the utilization of medical services, which factors are mainly affected by the inequality of medical service utilization in the same group. As the Chinese government’s core health policy to deal with the aging of the population and to achieve health for all [12], many studies have examined the role of family doctor contracted services in improving unhealthy lifestyles and preventing the incidence of chronic diseases [13, 14]. However, the impact of the use of contracted services by family doctors on eliminating inequality in utilization of medical services, especially the impact of eliminating inequality in utilization of medical services among the elderly, is rarely discussed. Therefore, this study took Shandong Province as the sample area and selected the elderly as a cross-sectional survey to explore the impact of family doctorcontract service system on the inequality in utilization of medical services of the elderly.

## Method

### Data sources

Shandong Province is located in eastern China and is one of the typical densely populated and economically developed regions in China. The economic development in the eastern, central and western regions of Shandong Province is unbalanced, the distribution of medical resources is unbalanced, and the population of elderly people ranks first in the country. To a certain extent, the utilization of family doctor contracted service in Shandong Province can be seen as a microcosm of the situation in China as a whole. Therefore, a thorough investigation of the degree of inequality in the utilization of family doctor contracted service for the elderly in Shandong province will provide general and representative data. The survey was conducted in March 2018, and the sampling method was multi-stage stratified random sampling. Taking into account the characteristics of economic development, geographic environment and health resources, three regions of Qingdao, Jining and Tai’an in Shandong Province are selected. 6 counties of each region are selected according to the characteristics of economic development and distance from urban areas. 3 towns are selected randomly of each county, with a total of 18 towns. 3 communities are randomly selected from each town, with a total of 54 communities. 20 elderly people who meet the sampling requirements are selected from the family doctor contracted service information database as the survey subjects, and plans to sample 1080 elderly.

Inclusion criteria for research subjects: (1) Age ≥ 65 years; (2) Have contracted with a family doctor for more than 1 year; Exclusion criteria: (1) Those who do not want to participate in the research; (2) Those who have not completed the questionnaire; (3) There is cognition impairment.

With the assistance of community (village) doctors, a questionnaire was conducted to collect the variables needed for the research. According to the criteria, 21 people refused to participate in the interview. Among the participants whom obtained informed consent from, 22 were excluded due to cognitive impairment (*n* = 13) and failure to complete the questionnaire (*n* = 9). Finally, 1037 valid questionnaires were obtained.

Variables for measuring the utilization of family doctor contracted service.

Based on the existing research and the actual utilization of the family doctor contracted service in China [15], four variables are used to measure the utilization of family doctor contracted service. (1) First consultation rate by family doctors: calculation formula: number of first consultation by family doctors in 1 month/number of visits in 1 month *100%; (2) physical examination rate: calculation formula = number of people who have received physical examination /research population*100%; (3) Healthy lifestyle guidance rate: calculation formula = number of people who have received healthy lifestyle guidance/ research population *100%; (4) chronic disease management rate: calculation formula = number of people included in chronic disease management/ The number of chronic patients among the research population*100%.

### Required Variables

We choose age, gender, whether there are chronic diseases and self-guided health rate as the necessary variables.

### Control variable

In order to better analyze the inequality of the utilization of family doctor contracted service for the elderly, this article includes a series of variables, including place residence, education level, marital status, family size (the number of family members living together), and economic status. In previous studies, personal income was mostly used to measure economic status. Since family doctor contracted service contracts with family as a unit, and the main body of utilization of medical service and health decision-making is often families rather than individuals, this article uses annual family income to measure individuals economic status. According to the method of five income groups, the economic status of the survey subjects are sorted from low to high, and divided into five groups of equal numbers, namely the poorest, the poorer, the general, the richer, and the richest.

### Measurement and Decomposition of Inequality

This article mainly uses the concentration index method proposed by Wagstaff to measure the inequality in the use of family doctor contracted service utilized by the elderly [16]. The concentration index is equal to twice the area defined as the area between the concentration curve and the 45° equality line. The concentration index ranges from − 1 to 1. In the absence of socioeconomic inequality, the concentration index is 0. The positive score of the concentration index shows the pro-rich health inequality, and the negative score shows the pro-poor health inequality. The greater the absolute value of the concentration index, the farther the concentration curve is from the 45-degree equality line, indicating the higher the degree of inequality represented by the corresponding variable. The concentration index (Eq.1) can be calculated using the following formula:

1 $$C=\frac{2}{u}\mathit{\operatorname{cov}}\left(y,r\right)$$

Among them, C represents the concentration index, cov is the covariance of y, μ represents the average value of the family doctor contracted service utilization index, y reflects the family doctor contracted service utilization index, and γ represents the score level of income distribution.

By grading the concentration index, it is possible to measure and explain the inequality of the utilization of medical and health services for the elderly. In this study, the method of decomposing the concentration index previously proved by Wagstaff et al. was used [17]. The contribution of individual factors to the concentration index is discussed, and the product of the sensitivity of health to individual factors and the degree of inequality in this factor is determined.

The linear additive regression of health (y) is expressed in Eq.2.

2 $${y}_i=\alpha +{\sum}_i{\beta}_j{x}_{ji}+{\sum}_k{\beta}_k{z}_{ki}+{\varepsilon}_i$$

In formula (2), *x*_*j*_ represents the required variable, and *Z*_*k*_ represents the control variable. *α*, *β*, and *ε* are constants, regression coefficients, and error terms, respectively.

The decomposition of the concentration index C could be specified as follows:

3 $$C={\sum}_j\left({\beta}_j{\overline{x}}_j/\mu \right){C}_j+{\sum}_k\left({\beta}_k{\overline{z}}_k/\mu \right)\;{C}_k{+}^G{C}_{\mu }{/}_{\mu }$$

In formula (3), *C*_*j*_ and *C*_*k*_ are the concentration indices of the required and controlled variables, respectively; ‾*x j* and ^*−*^*z*_*k*_ are the mean of *xj* and *z*_*k*_, respectively. The formula shows that the concentration index C of the utilization of basic medical and health services for the elderly is equal to the weighted sum of the concentration index of the need and control variables, and the product of the concentration index of each need and control variable and its weight is the inequality of the utilization of basic medical and health services. After eliminating the contribution of the required variable to inequality, the level of equity in the utilization of basic medical and health services can be calculated.

The decomposition of nonlinear models can only be achieved by linear approximation, which introduces errors and is very complicated. The existing literature shows that there is no significant difference in the decomposition of C when using linear and nonlinear models [18]. Therefore, this study uses a linear model to decompose the inequality of the utilization of medical and health services of the elderly.

### Analytical Method

The concentration curve was conveniently generated using MS Excel and the C and its decomposition computed using STATA12 statistical software.

## Results

Table 1 lists the basic situation of the participants. The majority of the samples are women (53.6%). More than half of the participants (56.25%) have an education level of elementary school and below. The average number of children of each participant is more than two. 79.88% of participants live with their family or other people. Nearly two-thirds of the participants have chronic diseases. Most elderly people (58.6%) think their health status is average. The average annual family income of the elderly is 31,474 yuan. It is worth noting that the standard deviation of annual income is large, indicating that the income gap between the elderly is also large.

**Table 1:** Description of the Basic Situation of the Participants

Variable	Mean (SD)/n(%)
Gender	
Male	478 (46.1%)
Female	559 (53.9%)
Age	
65–69	568 (54.8%)
70–79	380 (36.6%)
80 and older	89 (8.5%)
Education level	
Elementary school and below	583 (56.3%)
Middle School	373 (36.0%)
High school and above	80 (7.8%)
Family annual income	31,474 (33,063.6)
Number of children	2.46 (1.55)
Living situation	
Live alone	209 (20.1%)
Live with spouse/children	828 (79.9%)
Place of residence	
Urban	440 (42.4%)
Rural	607 (58.58%)
Chronic disease	
Yes	681 (65.7%)
No	356 (34.3%)
Self-evaluation of health condition	
Good	275 (26.5%)
Fair	608 (58.6%)
Poor	155 (14.9%)

Table 2 lists the utilization of contracted services for the elderly with different sociodemographic characteristics. From the perspective of the overall utilization of family doctor contracted service for the elderly, 24.6% of the people chose the family doctor for the first consultation, 65.8% of the people received the physical examination, 13.7% of the people received the healthy lifestyle guidance, and 52.2% of patients with chronic diseases are included in chronic disease health management. The poorer self-evaluation of health condition is, the higher the education level, and the higher the utilization rate of family doctor contracted service for elderly with chronic diseases. The rate of first consultation among rural residents is much higher than that of urban residents, and women’s healthy lifestyle guidance rate is 6.4% higher than that of men.

**Table 2:** Utilization of family doctor contracted service of the elderly with different sociodemographic characteristics (%)

	First consultation rate	Physical examination rate	Healthy lifestyle guidance rate	Chronic disease management rate	*P* value
Financial status					0.000
Poorest	20.1	70.1	12.7	50.2	
Poorer	30.6	72.6	11.2	54.1	
General	25.7	73.1	11.5	57.2	
Richer	13.2	60.7	13.1	53.1	
Richest	10.7	62.1	13.6	50.3	
Gender					0.000
Male	18.85	73.6	10.3	55.1	
Female	21.43	75.7	16.7	49.3	
Age					0.000
65–69	19.86	68.1	12.1	50.2	
70–79	21.05	65.6	15.6	53.1	
80 and older	19.26	67.2	12.3	57.4	
Education level					0.000
Elementary school or below	23.91	54.3	9.8	53.7	
Middle school	17.42	71.7	17.2	52.1	
High school or above	17.29	82.1	21.3	51.2	
Living situation					0.000
Live alone	20.02	70.3	15.6	54.1	
Live with spouse/children	20.72	60.4	9.8	50.3	
Place of residence					0.000
Urban	18.4	67.1	15.2	51.7	
Rural	30.3	63.6	10.2	52.7	
Chronic disease					0.815
Yes	36.66	66.2	16.7	71.3	
No	18.75	64.6	8.7	–	
Self-evaluation of health condition					0.000
Good	24.10	60.2	14.3	51.2	
Fair	24.80	64.1	13.2	51.7	
Poor	32.11	70.1	15.1	52.7	
Total	24.6	65.8	13.7	52.2	

Table 3 shows the concentration index of the utilization of family doctor contracted service in the elderly group. The concentration index of the first consultation rate and the healthy lifestyle guidance rate are 0.451 and 0.573, respectively. There is inequality in favor of the rich. On the contrary, the concentration index of health examination rate and chronic disease management rate is negative (− 0.260, − 0.518), which means an unfair situation to the poor. When comparing the absolute values of the concentration index of the four indicators, the absolute value of the healthy lifestyle guidance rate is the largest, indicating that the equality of the use of healthy lifestyle guidance may be the lowest. The absolute value of the physical examination is the smallest, indicating that the equality of the utilization of the physical examination may be the highest.

**Table 3:** Concentration Index of Utilization of Family Doctor Contracted Service of the Elderly

Variable	C	Std.	95%CI	
Family doctor contracted service utilization rate	0.451	0.0074	0.0794	0.1085
Physical examination rate	−0.260	0.0071	0.0327	0.0532
Healthy lifestyle guidance rate	0.573	0.0069	0.0444	0.0716
Chronic disease management rate	−0.518	0.0051	0.0552	0.0752

Table 4 describes the contribution of various factors to the inequality of the utilization of family doctor contracted service for the elderly. The main reasons for the unequal utilization of the first consultation service for the elderly are: education level, income, place of residence and self-evaluated health condition; the main reasons for the unequal utilization of the physical examination service for the elderly are: education level, income, place of residence and age; the main reasons for the unequal utilization of the health lifestyle guidance services for the elderly are: education level, income, place of residence and gender; the main reasons for the unequal utilization of chronic disease management services for the elderly are: education level, income, place of residence and living arrangement. Education level is the most important factor affecting the inequality of utilization of basic medical and health services for the elderly. Income is the second contributing factor leading to the inequality of the utilization of the first consultation rate among the elderly. The two contribution values are positive, indicating that the increase in income and education level has increased inequality in favor of the rich. Place of residence, self-guided health rate, and age contribute negatively to the inequality of the utiliozation of basic medical and health services, indicating that they affect the inequality of the elderly in the utilizationof basic medical and health services that favor the poor.

**Table 4:** Decomposition results of the contribution rate of each factor of the inequality of family doctor contracted service of the elderly

	First consultation rate		Physical examination rate		Healthy lifestyle guidance rate		Chronic disease management rate	
	Contribution	%	Contribution	%	Contribution	%	Contribution	%
Financial status (reference level = Poorest)		45.86		38.93		48.49		42.85
Poorer	0.0081	22.1	0.0027	6.14	0.0090	11.68	0.0071	10.55
General	0.0027	10.89	0.0033	7.24	0.0113	13.96	0.0082	11.88
Richer	0.0028	6.60	0.0025	7.89	0.0130	14.66	0.0069	10.34
Richest	0.0030	6.27	0.0032	7.66	0.0086	8.19	0.0063	10.08
Gender (reference level)								
Male		2.65		5.80		9.10		7.92
Female	0.0067	2.65	0.0075	5.80	0.0081	9.10	0.0086	7.92
Age (reference level)								
65–69		3.70		8.40		3.51		3.28
70–79	0.0011	1.68	0.0030	3.17	0.0051	5.34	0.0029	1.57
80 and older	0.0019	2.02	0.0047	5.23	−0.0026	−1.83	0.0035	1.71
Education level (reference level)								
Elementary school or below		52.2		55.21		56.10		57.81
Middle school	0.0142	21.29	0.0160	34.24	0.0152	24.75	0.0260	32.35
High school or above	0.0418	30.91	0.0104	20.97	0.0258	31.45	0.0176	25.46
Living situation (reference level)								
Live alone)		−4.38		−10.89		−5.17		−13.15
Live with spouse/children	−0.00638	−4.38	− 0.0070	− 10.89	0.0046	− 5.17	− 0.0118	− 13.15
Place of residence (reference level)								
Urban)		−17.95		−4.33		−11.73		−13.97
Rural	−0.0114	−17.95	− 0.0027	−4.33	− 0.0085	−11.73	−0.0102	− 13.97
Chronic disease (reference level)								
Yes)		7.35		5.22		7.30		9.83
No	0.0091	7.35	0.0051	5.22	0.0062	7.30	0.0076	9.83
Self-evaluation of health condition (reference level)								
Good		10.57		−2.60		−7.60		5.43
Fair	0.0062	6.14	0.0036	−1.36	0.0016	−2.23	0.0029	2.57
Poor	0.0053	4.44	0.0010	−1.24	0.0037	−5.37	0.0023	2.86

Table 5 describes the horizontal inequity index of utilization of family doctor contracted service of the elderly group after eliminating the influence of the required variable, which are 0.355,-0.229, 0.460and − 0.439, respectively. The results show that the required variable has little effect on the utilization of basic medical services for the elderly, and the horizontal inequity of the healthy lifestyle guidance rate is still the highest.

**Table 5:** Horizontal inequity in the utilization of family doctor contracted services in elderly households

	Family doctor first consultation rate	Physical examination rate	Health lifestyle guidance rate	Chronic disease management rate
Required variables	0.096	−0.031	0.113	−0.079
Control variables	0.336	−0.157	0.439	−0.403
Residual	0.019	−0.072	0.021	0.036
Total	0.451	−0.260	0.573	−0.518
Horizontal inequity	0.355	−0.229	0.460	−0.439

## Discussions

This article attempts to use the concentration index decomposition method for the first time to explain the inequality in the utilization of family doctor contracted service in the elderly population in China. This can not only help us find the root cause of the inequality in the utilization of family doctor contracted service of the elderly, but also has great significance for the implementation of the Chinese family doctor contracted service system and the achievement of the national strategy of achieving primary health care for all.

The descriptive results of this article first show that the utilization rate of physical examination of the elderly is the highest, at is 65.8%, which is much higher than the physical examination rate before the implementation of the family doctor contracted service system, and is also much higher than the physical examination rate for Chinese adults in the same period [19–22]. The rapid rise is caused by the free provision of physical examination services for the elderly [23], Since the implementation of the family doctor contracted service system, the first consultation rate of family doctors among the elderly has continued to rise, and is higher than the first consultation rate of the entire population. The elderly residing in rural areas are more inclined to make first consultation by family doctors than elderly residing in urban areas. Since China currently does not implement mandatory family doctors’ first consultation, due to the pursuit of better quality of medical services, the elderly in urban areas have a wider choice of options, so that family doctors have a smaller chance to be chosen for first consultation. Only 13.7% of the elderly receive healthy lifestyle guidance. Although the healthy lifestyle guidance service is also a free service, the utilization rate has not changed much since the implementation of the system. The possible reason is that the number of family doctors is in short supply, the workload is large, and there is not enough time and energy to complete these personalized services for each resident. Moreover, the state has not proposed detailed work standards and incentive plans for this service, and family doctors are not very restrictive and enthusiastic in providing this service.

The education level has increased the inequality in the utilization of basic medical and health services. This conclusion is consistent with the conclusions of some current researches on unequal utilization of medical services. Education level shows a positive correlation with the utilization of family doctor contracted service, and the decomposition of the utilization of family doctor contracted service inequality shows that education level has the first positive contribution to measuring inequality. In this study, the level of education exceeds income, becoming the primary factor that affects the measurement of inequality, and this result is still benefited from the free provision of family doctor contracted services. Secondly, in China, more than half of the family doctor contracted service projects for the elderly are services of a preventive health care nature. The education level is directly related to health awareness. The education level affects the elderly’s awareness of disease prevention and the contracted services [24]. It also affects the degree of trust in family doctors, and affects the ability of doctors to obtain, process and understand medical information in communication between doctors and patients. This leads to inequality in the utilization of health services by the elderly. This situation is more obvious in the utilization of healthy lifestyle guidance services.

Although income is an important factor that affects the utilization of basic medical and health service of the elderly, it is not the most important factor. This is inconsistent with the conclusions of most previous health service utilization equity literature [25, 26]. But this also precisely reflects the role of family doctor contracted service in alleviating the inequality in the utilization of health services for the elderly. This discovery is mainly due to free contracted service items and flexible service methods. The family doctor contracted service projects for the elderly are provided free of charge. These free projects have greatly increased the enthusiasm of the elderly in the utilization of health services. For the elderly with limited mobility, family doctors also provide flexible medical treatment methods in accordance with government subsidy regulations, such as on-site services, telephone consultations, and services by appointments, which increase the opportunities for the elderly to utilize health services. It shows that good public health policies can effectively improve the inequality in the utilization of physical examination services.

Another factor that affects the equity of utilization of basic medical and health service of the elderly is the living situation. It is generally believed that living with the elderly is more conducive for their children to care and support, and therefore has a positive effect on the utilization of medical services for the elderly. However, the results of this study show that compared with the living with their children, living alone has a significant positive impact on the health of the elderly. The possible explanation is that the current family resource allocation is generally leaning towards children. When a family has both elderly and young kids, the resources are usually allocated to children, thus the elderly may not be able to get enough care. When the elderly lives with their children, interpersonal relationships will also become complicated. The stimulation of the family environment on physical and mental health will be reflected in the health level [27].

Our research results further highlight the existence of regional differences. Comparing the concentration index between urban and rural areas, pro-rich inequality in rural areas is more obvious than that in urban areas. Although inequality of utilization of medial and health services exists in both urban and rural middle-aged and elderly groups, the degree of inequality in rural areas is higher than that in urban areas. Generally speaking, the inequality of the utilization of medical and health services in the urban and rural elderly groups is mainly caused by the imbalance of resource input and the uneven distribution of health resources under the urban-rural dual structure [28]. Although health facilities in many rural areas have improved in recent years, these urban-rural gaps still exist. Therefore, narrowing the gap between urban and rural areas is conducive to the utilization of family doctor contracted service by the elderly.

## Conclusion

The family doctor contracted service has had a positive impact on alleviating the inequality of the utilization of basic medical and health services of the elderly, but the simple increase in the number of contracts cannot achieve the expected goals of the policy. The implementation of the family doctor contracted service system has shown positive effects in improving the physical examination and chronic disease management of the elderly. There is still inequality of “pro-rich people” in the first consultation by family doctors. Due to the free provision od basic medical services, income is no longer the most important factor affecting the utilization of basic medical and health services for the elderly. Social and human factors such as education level, living arrangement and place of residence become the main factors. In order to further promote the equality of the utilization of basic medical and health services for the elderly, the cooperation between the health system and other social and economic systems should be strengthened to enhance the health literacy of the elderly, to narrow the gap between the rich and the poor, and to build a harmonious family relationship.

### Limitations

Firstly, the data in our study are only from Shandong Province, which may limit the generalizability of the research results in other regions. Secondly, due to the availability of data, this study did not consider all the factors that may affect the utilization of family doctor contracted service, such as the perception of the elderly towards family doctor contracted service, and the degree of knowledge about the family doctor contracted service. Thirdly, since this study is a cross-sectional study. we can’t to continuously observe and compare the utilization of family doctor contracted services in the elderly. It is recommended to conduct continuous observation in the sample area.

## References

[1] World Health Organization. World report on aging and health. World report on ageing and health (who.int).

[2] National Bureau of Statistics of China. Statistical Communiqué of the People’s Republic of China on the 2019 National Economic and Social Development. Statistical Communiqué of the People's Republic of China on the 2019 National Economic and Social Development (stats.gov.cn).

[3] Sanxiu W (2016). Active aging and medical precise protection in health poverty and older people. China Med Insur.

[4] Li Z, Song Lin WANG, Jing ZHAO (2019). Hotspots and emerging trends in contracted family doctor services research:a scientometric analysis in CiteSpace. Chinese Gen Pract.

[5] ShanShan L, Yan L, Tao Z (2021). The developing family doctor system: evidence from the progress of the family doctor signing service from a longitudinal survey (2013–2016) in Pudong New Area, Shanghai. BMC Family Practice.

[6] Zhou J, Peng Y, Zhang Z (2020). Research on the Performance and Trust Status Parties in Contract Services of Family Doctors in the Suburbs of Beijing. BMC Health Serv Res.

[7] Rueda S (2012). Health inequalities among older adults in Spain: The importance of gender,the socioeconomic development of the region of residence, and social support. Women’s Health Issues.

[8] Rueda S, Artazcoz L, Navarro V (2008). Health inequalities among the elderly in Western Europe. Epidemiol Community Health.

[9] Park EJ, Cho SI, Jang SN (2012). Poor health in the Korean older population: Age effect or adverse socioeconomic position. Arch Gerontol Geriatr.

[10] Braveman P (2006). Health disparities and health equity: Concepts and measurement. Annu Rev Public Health.

[11] Lowry D, Xie Y. Socioeconomic Status and Health Differentials in China: Convergence Or Divergence at Older Ages? Popul Stud Center. 2009.

[12] Boao Health Forum. Achieving health for all. https://baijiahao.baidu.com/s?id=1636221408440089027&wfr=spider&for=pc.

[13] Vogt F, Hall S, Marteau TM (2005). General practitioners' and family physicians' negative beliefs and attitudes towards discussing smoking cessation with patients: A systematic review. Addiction.

[14] Beich A, Gannik D, Malterud K (2002). Screening and brief intervention for excessive alcohol use: Qualitative interview study of the experiences of general practitioners. BMJ.

[15] Diehr P, Yanez D, Ash A, Hornbro M, Lin DY (1999). Methods for Analyzing Health Care Utilization and Costs. Ann Rev PublicHealth.

[16] Wagstaff A, van Doorslaer E (2000). Measuring and testing for inequity in the delivery of health care. J Hum Resour.

[17] Wagstaff A, van Doorslaer E, Watanabe N (2003). On decomposing the causes of health sector inequalities with an application to malnutrition inequalities in Vietnam. J Econ.

[18] Memirie ST, Verguet S, Norheim OF, Levin C, Johansson KA (2016). Inequalities in utilization of maternal and child health services in Ethiopia: the role of primary health care. BMC Health Serv Res.

[19] Zhu Q, Han PF (2012). Results of Physical Examination of Over - 65 - year - old Elderly in A Community of Shanghai. Chinese General Practice.

[20] Wang Q, Zhang YY, Chen P (2014). The situation of periodic check up for the elderly people in community. Chinese General Practice.

[21] Chen R, Shan shan Liu, Hua Yan, et al. (2021). Health Behaviors Among Residents in Pudong New Area. Chinese Primary Health Care.

[22] Lu Z, Wei C, Zhu LC (2020). Status and influencing factors of health satisfaction and attitude towards shared medication among community residents in Wuxi city. Chin Public Health.

[23] Liu Y, Kong Q, Wang S (2020). The impact of hospital attributes on patient choice for first visit: evidence from a discrete choice experiment in Shanghai, China. Health Policy Plann.

[24] Weijing D, Yinghua L, Xueqiong N (2015). Analysis of status and influence factors of health literacy of Chinese residens aged 60-69. China’s Health Educ.

[25] Doorslaer EV, Koolman X (2004). Explaining the differences in income-related health inequalities across European countries. Health Econ.

[26] Vincent SS (2016). Socio-economic inequalities and their impact on health in Pakistan. Int J Res Nurs.

[27] Long Y, Lydia WL (2016). “How Would We Deserve Better?” Rural-Urban Dichotomy in Health Seeking for the Chronically Ill Elderly in China. Qual Health Res.

[28] Chenjing F, Ouyang W, Tian L (2019). Wensheng Miao. Elderly Health Inequality in China and its Determinants: A Geographical Perspective. Int J Environ Res Public Health.

